# Ossification of the pseudarthrosis following the Sauvé-Kapandji procedure: a case report and review of the literature

**DOI:** 10.1080/23320885.2021.1910040

**Published:** 2021-04-16

**Authors:** Jesse Seilern und Aspang, David Böckmann, Jochen Erhart, Thomas Haider

**Affiliations:** a Department of Orthopaedics and Trauma-Surgery, Medical University of Vienna, Vienna, Austria; b Department of Orthopaedics and Traumatology, Brothers of Saint John of God Eisenstadt, Eisenstadt, Austria

**Keywords:** Sauvé-Kapandji procedure, pseudarthrosis ossification, ulnar synostosis, revision surgery, IV – V

## Abstract

We report a case of new onset pain and loss of forearm rotation 3 years after Sauvé-Kapandji (SK) procedure. A revision ulnar osteotomy with application of bone wax restored ROM through 17 months follow-up. A literature review of pseudarthrosis ossification after SK procedure was also performed.

## Introduction

The distal radioulnar joint (DRUJ) accommodates several culprits for orthopaedic ailments, including inflammatory arthritis, osteoarthritis, post-traumatic derangements and congenital deformities. Pain related to isolated joint instabilities after ligament rupture, triangular fibrous complex injuries or ulnar impaction syndrome are also common reasons for the surgical approach of the DRUJ after failed conservative treatment [1].

Given this broad spectrum of pathologies and lesions, a variety of salvage procedures has been described. As early as 1855, Malgaine defined the resection of the ulnar head, which was later popularized by Darrach [2]. In 1936, Sauvé and Kapandji described a technique involving DRUJ arthrodesis and formation of an ulnar pseudarthrosis proximal to the DRUJ [3]. The latter was performed by Baldwin in 1921 in a successful attempt to preserve supination and rotation in malunited distal radius fracture [4]. The SK procedure has since been modified amid critical analysis of long-term outcome studies [5].

Despite universally acknowledged favorable outcomes of this technique, frequent surgical complications have been noted throughout historical data, including non-union or delayed union of the arthrodesis, painful instability of the proximal ulnar stump, and fibrous or osseous union at the pseudarthrosis site [6].

A substantial amount of qualitative investigations has fostered surgical modifications and treatment algorithm adjustments to accommodate the first two complications [7]. However, ossification of the pseudarthrosis, resulting in debilitating decreased range of motion, pain, and possibly requiring revision surgery is inconsistently described among failure rates across the current body of literature; and when encountered, vaguely characterized. To our knowledge, no investigation has assessed this complication in detail, since originally mentioned by Sanders et al. [8].

Therefore, the aim of this study is to present a clinical case of ossification of the pseudarthrosis and review of the literature in order to seek clarification concerning the influences and surgical background of this particular complication after the SK procedure.

## Case

**Figure 1.**
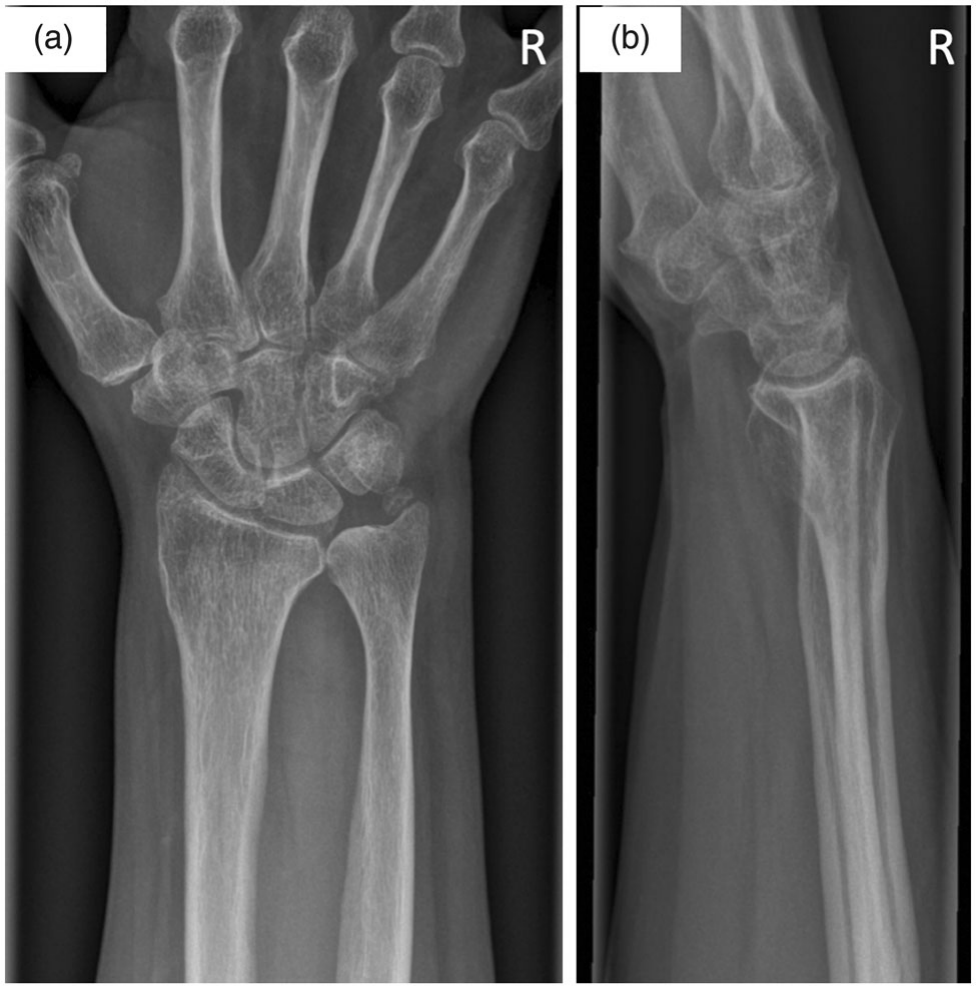
Plain AP (a) and lateral (b) radiographs of the right wrist on initial presentation demonstrate distal radioulnar joint arthrosis, positive ulnar variance, and ossicle formation near the tip of the ulnar styloid.

**Figure 2.**
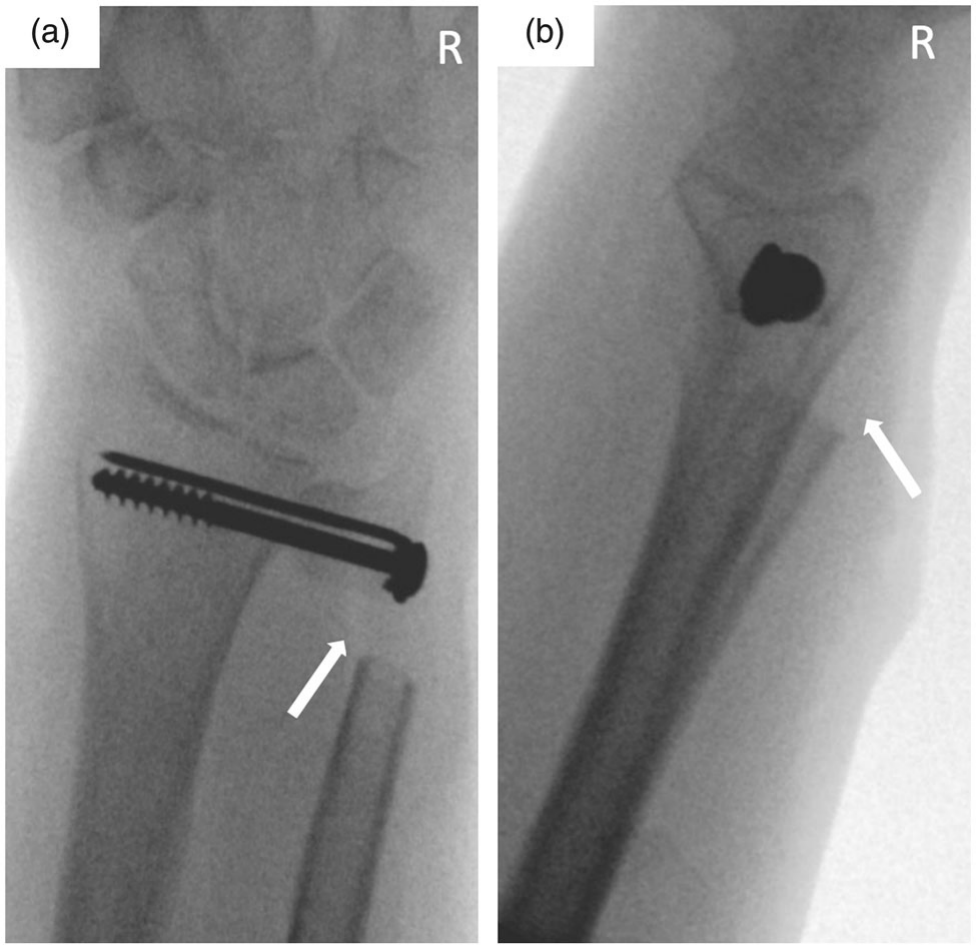
Intraoperative fluoroscopic AP (a) and lateral (b) images of the right wrist at index surgery (Sauvé-Kapandji procedure) with arthrodesis using a cancellous bone screw and K-wire. Note the clean ulnar gap without evidence of remaining osseous particles (white arrow).

Written informed consent was obtained from the patient for their anonymized information to be published in this article. A 47-year old right-hand dominant female patient initially presented to our institution with ongoing pain and limited range of motion of her right wrist. She reported onset of symptoms after a minor trauma 6 months prior. Repeated conservative treatment including physiotherapy, infiltrations, and oral pain medication did not provide lasting pain relief. On presentation, the patient complained about ulnar-sided wrist pain with limited pro- and supination to 20°-0°-20° and unrestricted extension and flexion. Plain film radiographs were obtained showing DRUJ arthrosis, a positive ulnar variance, dorsal ulnar subluxation, and ossicle formation near the tip of the ulnar styloid (Figure 1). Due to persisting limitation of pro- and supination we recommended a Sauvé-Kapandji procedure. The patient gave her informed consent and surgery was performed without complications (Figure 2 *– intraoperative images*).

The procedure was performed under general anesthesia and pneumatic tourniquet with the patient in supine position. A curved skin incision was made over the fifth extensor compartment. Care was taken not to damage the dorsal sensory branch of the ulnar nerve. After subluxation of the extensor digiti minimi tendon, the DRUJ was opened and the preparation was carried out more proximally to facilitate the planned osteotomy. Care was taken to preserve the periosteum before resecting 7 mm of the subcapital ulnar shaft under fluoroscopic control and the remaining periosteal ulnar flap was sutured together as an interposition to stabilize the proximal ulna. The distal ulnar head and radial fovea was prepared with a drill burr to facilitate bony fusion. Finally, the arthrodesis of the DRUJ was performed with a 3.5 mm lag screw and 1.8 mm k-wire. After hemostasis, the extensor digiti minimi tendon remained subcutaneously and the wound was closed using interrupted sutures. No suction drain was used. Postoperatively, a below the elbow splint was applied for 6 weeks and early active and passive forearm rotation was performed.

**Figure 3.**
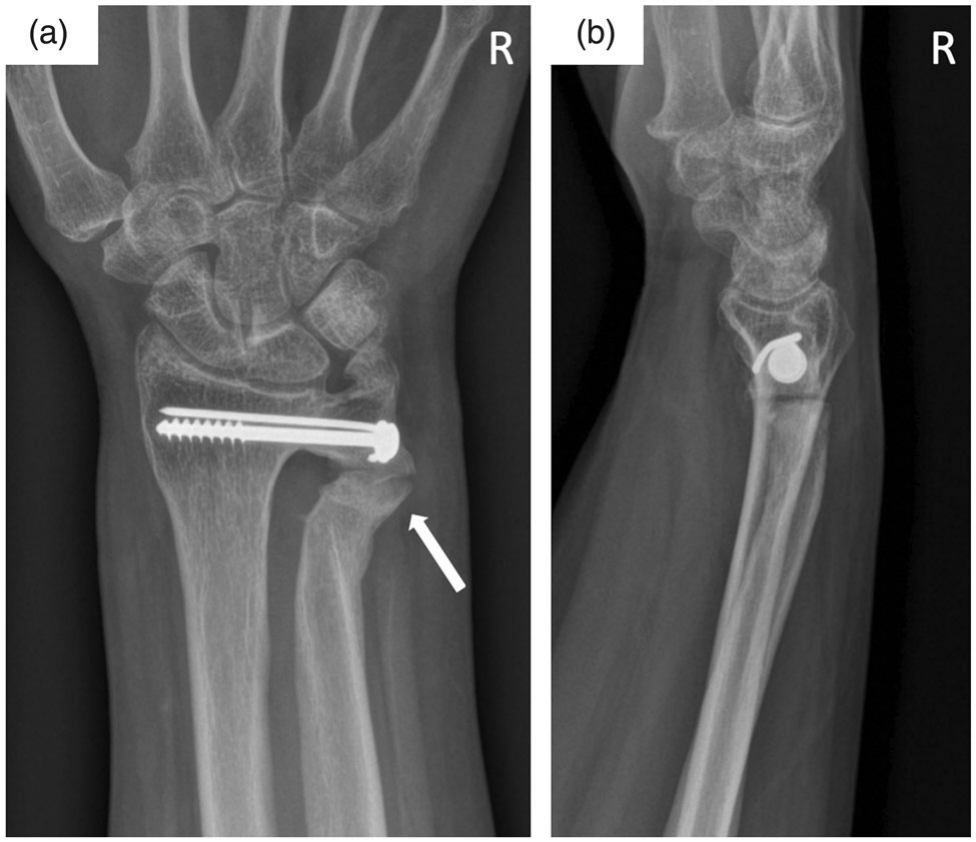
Follow-up AP (a) and lateral (b) radiographs of the right wrist 18 months after index surgery demonstrating bony consolidation of the distal radioulnar joint. Note the prominent ossification of the ulnar gap pseudarthrosis site (white arrow).

The patient returned to our office 18 months later due to pain and returning limitation of forearm rotation, while she had reported improvement of her symptoms with no limitation of motion postoperatively, with a new onset of symptoms about 1 month prior to the visit. Obtained radiographs exhibited formation of a pseudarthrosis within a prominent ossification at the ulnar osteotomy site (Figure 3). Forearm rotation was again limited to 15°-0-15°. The patient gave her consent for revision osteotomy of the pseudarthrosis.

**Figure 4.**
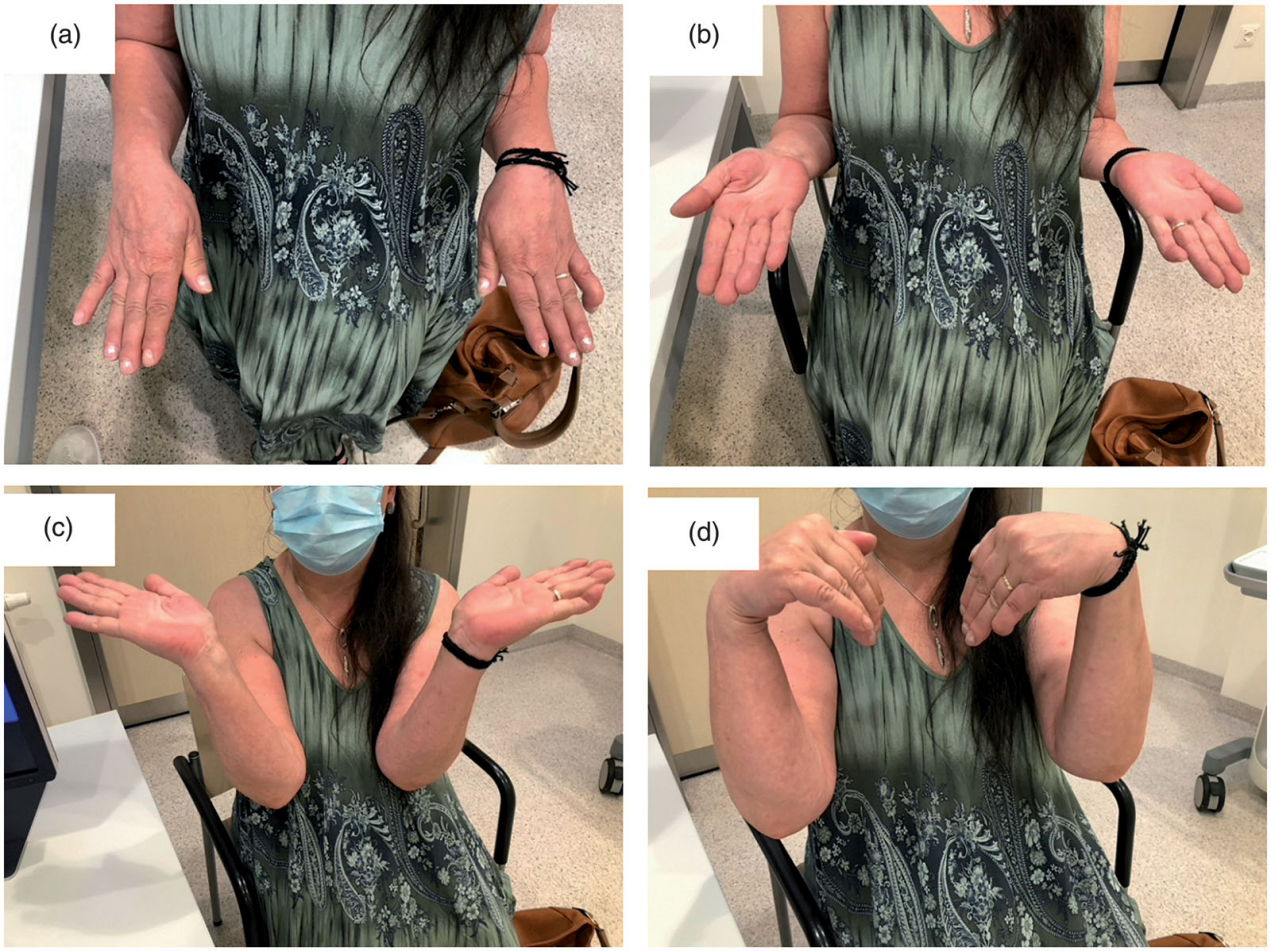
Clinical images 17 months after revision surgery with 15° pronation deficit (a), 15° supination deficit (b), full extension (c) and 15° flexion deficit (d) of the right wrist.

**Figure 5.**
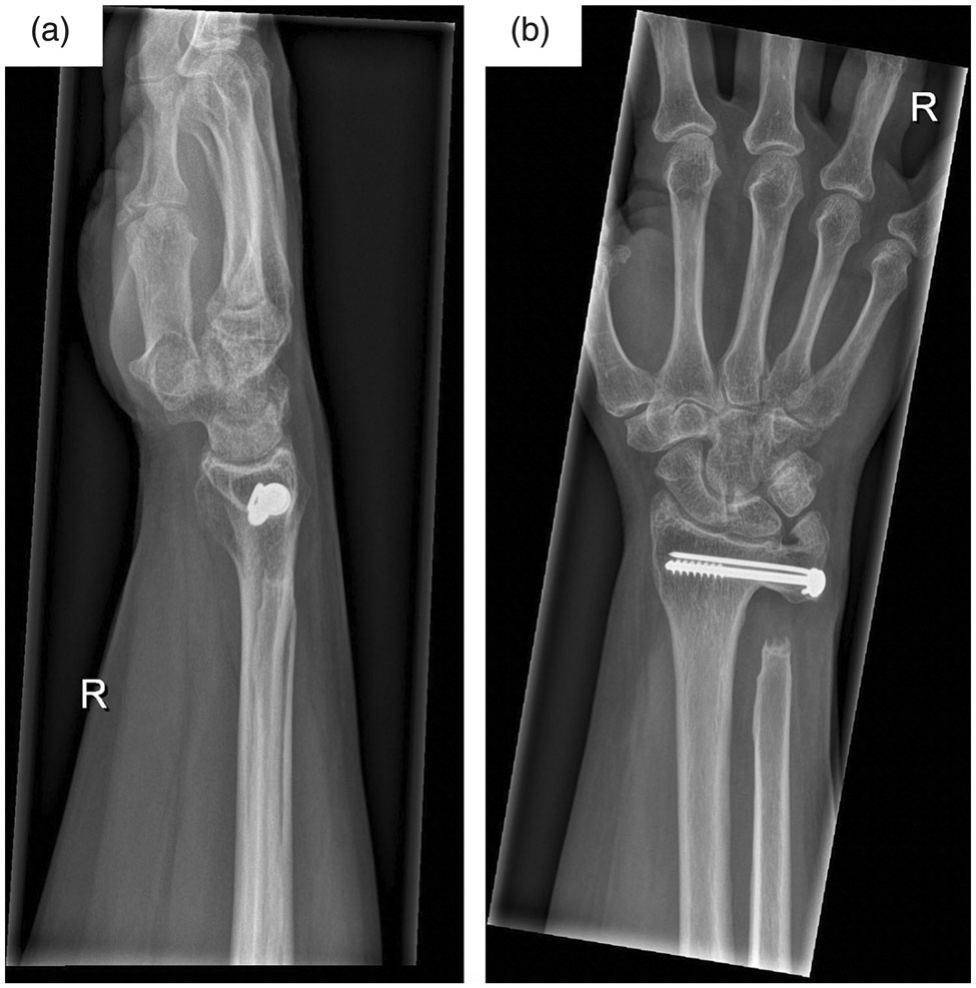
Follow-up AP (a) and lateral (b) radiographs of the right wrist 17 months after revision osteotomy of the ulnar pseudarthrosis ossification. Note the clean ulnar gap without evidence of remaining osseous particles (white arrow).

For the revision surgery the prior approach was used, and the proximal scar was extended. The ossification and fibrous scar tissue at the new formed pseudarthrosis were resected using a rongeur and the osteotomy was carried out in the extraperiosteal plane. Additionally, bone wax was used to promote homeostasis and a suction drain was put in place before closure of the wound. No further splint was used, and active range of motion was promoted under supervision starting right after surgery. At final follow-up 17 months after revision surgery, the patient remained free of pain and able to demonstrate acceptable range of motion, with a 15° deficit in wrist flexion, supination and pronation (Figure 4). Radiographic and clinical outcome demonstrated acceptable bony alignment (Figure 5) and Disabilities of the Arm, Shoulder and Hand Score, Mayo wrist score and patient rated wrist evaluation of 7, 95, and 19, respectively.

## Discussion

Among various salvage procedures of the distal radioulnar joint (DRUJ), the Sauvé-Kapandji (SK) procedure has been labeled as superior, also in young and active patients [9]. Most notably, the creation of an ulnar pseudarthrosis proximal to the fusion site has the potential to fully restore limited or lost forearm rotation. Yet, ossification of this pseudarthrosis and eventual synostosis of the ulnar gap inevitably reverses regained forearm rotation and oftentimes requires surgical intervention. We found 24/245 (9.8%) documented cases describing ossification of the pseudarthrosis, of which 16 (66.6%) underwent revision surgery within the documented period (Table 1).

**Table 1.** Overview and characteristics of included studies in the literature review.

Author	Surgical technique^a^	Ulnar resection in mm	Mean time to ossificatio^b^ (range)	Ossification of pseudarthrosis	Revision surgery (total)
Giberson-Chen et al. [17]	PQ interposition only ECU tenodesis or both	10	24 (12–108)	4/57 (7%)	Osteotomy ± HWR x1 (4)
Verhiel et al. [15]	PQ interposition + ECU tenodesis PQ interposition + FCU tenodesis PQ interposition + combined FCU-ECU tenodesis	n.a.	14	5/28 (18%)	Osteotomy (3)
Tomori et al. [10]	PQ interposition only PQ interposition + ECU suspension PQ interposition + ECU tenodesis	15	16 (8–35)	1/38 (3%)	Osteotomy + PL tendon interposition (1)
Arora et al. [18]	PQ interposition	n.a.	46 (16–58)	1/11 (9%)	Osteotomy + HWR (1)
Jacobsen et al. [12]	PQ interposition	15	67 (60–97)	1/20 (5%)	(0)
Low and Chew [9]	PQ interposition + ECU tenodesis	10	32,8 (22–48)	1/16 (6%)	n.a.(1)
Lamey and Fernandez [5]	PQ interposition + FCU tenodesis	n.a.	12 (5–21)	4/18 (22%)	Osteotomy (1)
Zachee et al. [14]	PQ interposition	15	17,8 (3–48)	4/31 (10%)	Osteotomy(4)
Nakamura et al. [13]	PQ interposition	15	28 (12–57)	1/15 (7%)	n.a. (1)
Sanders et al. [8]	PQ interposition	15	26–28	2/11 (18%)	(0)

PQ: pronator quadratus muscle; ECU: extensor carpi ulnaris muscle; FCU: flexor carpi ulnaris muscle; PL: Palmaris longus muscle; HWR: hardware removal; ^a^Sauvé-Kapandji procedure with varying modifications addressing the proximal ulnar stump; ^b^time to ossification after index surgery.

A variety of surgical modifications of the SK procedure have been labeled to ameliorate the complication of ulnar stump pain- and hypermobility [10]. However, while ossification of the pseudarthrosis produces a similar debilitating complication rate amounting in surgical revision, above mentioned surgical modifications do not seem to affect the occurrence rate (Table 1). Moreover, the opposing spectrum of proximal ulnar stump mobility and secondary closing of the pseudarthrosis gap *via* ossification should be held in consideration. While the pseudarthrosis may undergo ossification, the proximal stump is arguably secondarily stabilized and therefore could decrease the risk of hypermobility, which is the culprit of the most common complications, namely radioulnar convergence and ulnar stump pain.

Nevertheless, only few considerations for surgical adjustments to decrease the risk of ossification can be cited in historical data. Lluch et al. [6] argue that the best results will be obtained if the pseudarthrosis is done at the level of the ulnar head, removing only 5 mm of bone and Daecke et al. [11] suggest keeping the gap narrow to prevent proximal ulnar stump hypermobility. Contrarily, several authors argue that a gap of up to 15 mm produces most favorable results [8,10,12,13] (see Table 1), and Zachee et al. [14] state that early postoperative mobilization of the wrist could prevent re-ossification of the gap. In the current case, the surgeons resected 7 mm of subcapital ulnar shaft on the index procedure.

Verhiel et al. [15] comment that prior trauma and preparation of the DRUJ for fusion may create a stimulus for heterotopic ossification and that it is more likely to occur with incomplete resection of the ulnar periosteum, while there is also mention of an intraperiosteal resection technique [16], which leaves periosteal tissue in the pseudarthrosis gap. In our case, an intraperiosteal osteotomy was performed during index surgery, of which the remaining periosteum was utilized as interposition material. This is the first case at our institution, which demonstrated above mentioned complication with this technique.

Synostosis can develop fairly rapidly. The literature review showed a range of mean time to ossification of the pseudarthrosis of 12–67 months in the 24 (9.8%) of the total 245 cases (Table 1). Yet, these numbers do not provide detailed information on the complete bridging of the gap or synostosis, due to oftentimes limited radiologic data of the respective studies. In our case, radiologic evidence of ossification was noted 17 months after surgery and progressed precipitously to near complete synostosis within 18 months postoperatively with concomitant loss of pronation and supination. With the distal radioulnar arthrodesis-hardware out of reach of the proximal ulnar stump, the remaining potentially stimulating and modifiable factors at the osteotomy site is limited to soft tissue interposition-, stabilization, or suspension methods previously described by several authors (Table 1) [5,9,10,15,17]. However, according to this literature review, no significant pattern can be elucidated when evaluating for a trend of ossification towards one specific procedure modification. In our case, revision surgery was carried out with an osteotomy in the extraperiosteal plane with removal of the ossification and fibrous scar tissue. Furthermore, application of bone wax onto the ulnar osteotomy sites during revision surgery, may have minimized the risk of re-ossification. Bone wax is commonly utilized in orthopaedic surgery as a mechanical barrier to achieve haemostasis of the bone. To date, there is no data on its effects of limiting pseudarthrosis ossification and further observation within the scope of a case control study is warranted to assess whether bone wax application modifies the risk of pseudarthrosis re-ossification after revision surgery.

In the literature review, up to 66.6% (16/24) of the total cases with documented ossification of the pseudarthrosis received surgical treatment, with individual revision rates as high as 12.9% [14] (Table 1). This number is significantly higher than the most recently reported published revision rate [17] (7%). Still, not all cases with evidence of pseudarthrosis ossification exhibited debilitating symptoms or progression to synostosis [8].

The indication for revision surgery remained consistent across all listed investigations for rotatory deterioration. Lamney and Fernandez [5] described removal of hardware and excision of the revision osteotomy with tendon interposition of the palmaris longus tendon, although the outcome of both treatment options was not published. Alternative revision surgery options have been described to involve creating a one-bone forearm [19] or wide excision of the ulna [20]. Furthermore, Fok et al. [21] present a case series of patients treated with a spherical ulnar head prosthesis, who showed encouraging midterm results in all categories, ROM, grip power and the Disability of the Arm, Shoulder, and Hand Score (DASH). Overall, a common revision strategy for the indication of loss of forearm rotation due to ossification of pseudarthrosis or synostosis is inconsistently defined across literature, with the majority involving revision osteotomy [5,14]. In our case, the patient also received revision osteotomy in an extraperiosteal plane, and we presented the first documented application of bone wax to the ulnar stump, which showed no signs of re-ossification after 17 months follow-up. In conclusion, Potential risk factors of recurrent synostosis (ossification) remain undetermined throughout literature and qualitative investigations would be required to determine respective re-ossification patterns. However, revision osteotomy represents a popular option in these cases, and we present an acceptable outcome with an extraperiosteal revision osteotomy with additional application of bone wax to the ulnar stump.
